# Inhibition of hedgehog signaling improves the anti-carcinogenic effects of docetaxel in prostate cancer

**DOI:** 10.18632/oncotarget.2932

**Published:** 2015-02-17

**Authors:** Murielle Mimeault, Satyanarayana Rachagani, Sakthivel Muniyan, Parthasarathy Seshacharyulu, Sonny L. Johansson, Kaustubh Datta, Ming-Fong Lin, Surinder K. Batra

**Affiliations:** ^1^ Department of Biochemistry and Molecular Biology, University of Nebraska Medical Center, Omaha, NE, USA; ^2^ Department of Pathology and Microbiology, University of Nebraska Medical Center, Omaha, NE, USA; ^3^ Buffet Cancer Center, Eppley Institute for Research in Cancer and Allied Diseases, University of Nebraska Medical Center, Omaha, NE, USA

**Keywords:** Prostate cancer, hedgehog signaling pathway, GDC-0449, docetaxel, PC stem/progenitor cells and PC3 xenograft

## Abstract

The establishment of docetaxel-based chemotherapeutic treatments has improved the survival of castration-resistant prostate cancer (CRPC) patients. However, most patients develop resistance supporting the development of therapy. The current study was undertaken to establish the therapeutic benefit to target hedgehog signaling cascade using GDC-0449 to improve the efficacy of chemotherapeutic drug, docetaxel. Here, we show that the combination of GDC-0449 plus docetaxel inhibited the proliferation of WPE1-NB26 cells and PC3 cells *via* a blockade of G1 and G2M phases. The combined treatment significantly inhibited PC cell migration *in vitro*. Moreover, the apoptotic effect induced by GDC-0449 plus docetaxel on PC3 cells was mediated, at least partly, *via* the mitochondrial membrane depolarization, H_2_O_2_ production and caspase cascade activation. Interestingly, GDC-0449 was effective at inhibiting the prostasphere formation, inducing the prostasphere disintegration and apoptotic death of side population (SP) from PC3 cells and reversing the resistance of SP cells to docetaxel. In addition, GDC-0449 plus docetaxel also have shown a greater anti-tumoral growth inhibitory effect on PC3 cell xenografts. These findings support the use of the hedgehog inhibitor GDC-0449, which is currently in clinical trials, for improving the anticarcinogenic efficacy of docetaxel-based chemotherapeutic treatments against locally advanced, AI and metastatic PC.

## INTRODUCTION

Prostate cancer (PC) is the most commonly diagnosed cancer and the second leading cause of cancer-related deaths among United States men [1]. Organ-confined and locally advanced PC are managed well by radical prostatectomy, radiation and androgen deprivation therapy (ADT) [2–4]. However, over a median period of 18–24 months, most tumors eventually relapse and develop into castration-resistant PC (CRPC) [5, 6]. The current first-line docetaxel-based chemotherapeutic treatment regimens for metastatic CRPC are only palliative with limited survival rate [7]. Moreover, acquired resistance to docetaxel is associated with a poor prognosis and limited treatment options [6, 8]. Therefore, new treatment options are being actively pursued with additional agents or in combination with the existing chemotherapeutic agents to extend the survival of metastatic CRPC patients.

Numerous investigations have revealed that the sustained activation of distinct growth factor pathways, including sonic hedgehog cascade, may contribute to the proliferation, survival and therapeutic resistance of PC cells [9–11]. More specifically, the enhanced expression of hedgehog signaling elements, including sonic hedgehog ligand (SHH) and glioma-associated oncogene homolog-1 (GLI-1) that acts as a zing finger transcription factor, has been observed in PC epithelial cells and/or stromal compartment of tumors during disease progression to aggressive, metastatic and AI PC [12–18]. It has also been shown that the inhibition of the hedgehog cascade, by using either an anti-SHH antibody or a specific inhibitor of smoothened (SMO) activity, such as cyclopamine, suppressed the growth and induced the apoptosis of PC cells *in vitro* and *in vivo*, whereas normal prostate epithelial cells were insensitive to the cytotoxic effects of these agents [14, 15, 19, 20]. Moreover, our previous results have indicated that the blockade of the hedgehog and EGFR pathways using cyclopamine and gefitinib induced the anti-proliferative and apoptotic effects and improved the cytotoxic effect of docetaxel on androgen-dependent (AD), androgen-independent (AI) and metastatic PC cell lines [15, 19]. Additionally, recent preclinical studies and clinical trials have also revealed that the targeting of the hedgehog signaling pathway with GDC-0449 (vismodegib) induced the anticarcinogenic effects against aggressive cancers, including patients with medulloblastoma, breast, lung and basal cell carcinomas without major systemic toxicity [21–26].

Accumulating lines of evidence have also indicated that cancer stem/progenitor cells, also designated as cancer- or tumor-initiating cells (TICs), can contribute to the initiation and progression of solid tumors [27–30]. Particularly, it has been shown that PC stem/progenitor cells endowed with stem cell-like properties play a crucial role both in the initiation and progression of PC [31–34]. The PC stem/progenitor cells that are resistant to conventional radiation, ADT and docetaxel-based chemotherapy may be responsible for the tumor re-growth and disease relapse [35–37]. The SHH signaling pathway plays key roles in the regulation of the embryonic development and normal physiological process, such as the proliferation, differentiation and survival [38]. Consequently, the deregulation of SHH signaling cascade often results in the cancer development [39, 40]. In this regard, it has been shown that the SHH signaling pathway is aberrantly activated in PC [12] and plays a significant role in the regulation of self-renewal, differentiation and survival of PC cells. Altogether, these studies suggest that the blocking of the SHH-GLI signaling pathway will provide an attractive therapeutic strategy to treat advanced and metastatic PC caused by the prostate stem/progenitor cells and prevent disease relapse.

In the present study, *in vitro* and *in vivo* studies have been undertaken to test the hypothesis that the inhibition of the SHH signaling pathway would enhance the anticarcinogenic activity of docetaxel on CRPC. The results have indicated that GDC-0449, which specifically targets the SHH pathway, inhibited both *in vitro* and *in vivo* proliferation of PC cells. In addition, GDC-0449 was also effective at enhancing the apoptotic effect of docetaxel in PC cells. Importantly, GDC-0449 also inhibited the self-renewal of side population (SP) PC-3 cells expressing higher levels of stem-cell markers, and which have been implicated in promoting epithelial-mesenchymal transition (EMT) process and drug resistance. Taken together, the results have shown the potential benefit to use GDC-0449 for inducing anti-proliferative, anti-invasive and apoptotic effects and improving the cytotoxicity induced by current chemotherapeutic drug, docetaxel on AI PC cells, including SP cells with stem cell-like properties.

## RESULTS

### Expression levels of SHH and GLI-1 in normal prostate and adenocarcinoma of human prostate tissue specimens

**Figure 1:**
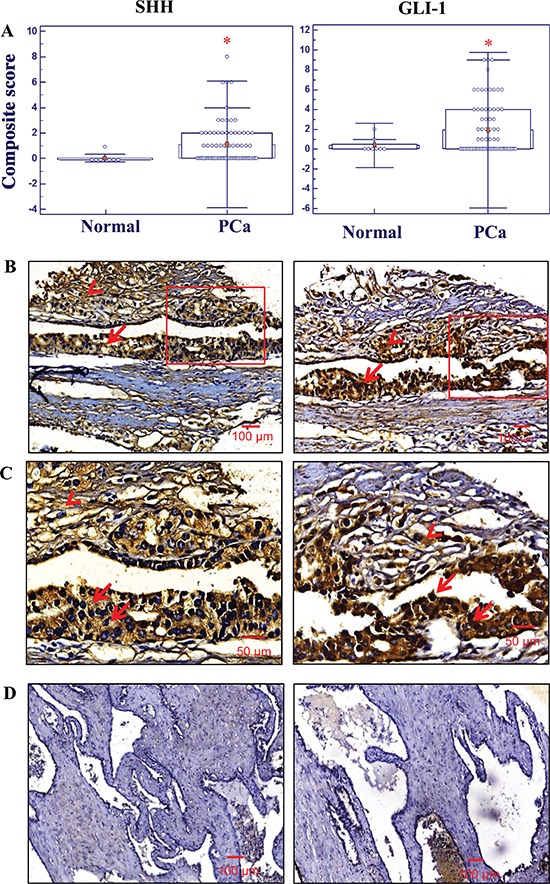
Immunohistochemical analyses of the expression levels of sonic hedgehog (SHH) and glioma-associated oncogene homolog-1 (GLI-1) in normal prostate and prostatic adenocarcinoma tissues Immunohistochemistry (IHC) staining was performed in tissue microarrays using specific antibodies against SHH and GLI-1 as indicated in the section of materials and methods. **(A)** Composite scores of expression levels of SHH and GLI-1 in normal prostate and PC tissue specimens. IHC analyses shows higher levels of SHH and GLI-1 expression in prostate adenocarcinoma tissues (*n* = 76) (*p < 0.0005) when compared to normal tissues obtained at autopsy of different ages (19–43 years) (*n* = 8). **(B)** Representative micrograph of SHH and GLI-1 expression in stromal and epithelial cells of human prostatic adenocarcinoma tissues. Arrow indicates the positive immunostatining for cytoplasmic SHH and nuclear GLI-1 expression. **(C)** Higher magnification of SHH and GLI-1 positive cells. Representative tissue sections from prostate adenocarcinoma were used for SHH and GLI-1 comparison. **(D)** In normal prostate tissues, both SHH and GLI1 protein expression was comparatively lower than in prostate adenocarcinoma cells. All the micrographs are in the same magnification (×100); scale bar: 100 μm, except higher magnification (×200); scale bar: 50 μm.

Identical tissue arrays containing 76 human PC specimens with 8 normal tissue specimens were stained for both SHH and GLI1 protein expression by immunohistochemical technique. The SHH-positive detection rate was 46% for 76 cases of the prostate carcinoma specimens (Gleason scores:6–10; stages T2-T4), and the mean of composite score values for SHH expression was statistically higher (*p < 0.0002) for PC tissues (1.0 ± 0.2) when compared to normal prostate tissue specimens (0.1 ± 0.1) (Figure 1A). Similarly, an enhanced expression of the transcription factor of the hedgehog cascade, GLI-1 was also observed in 47% of 76 cases of prostatic adenocarcinomas. The mean of composite score values obtained for GLI-1 expression in malignant epithelial cells in prostatic adenocarcinoma specimens (1.9 ± 0.3) was significantly higher as compared to the value for normal tissues (0.4 ± 0.3; *p < 0.0005) (Figure 1A). More particularly, the results of immunohistochemical analyses have indicated that an enhanced expression of SHH ligand primarily occurred in the cytoplasm (indicated by the arrow) of the malignant epithelial cells (Figures 1B and 1C) as compared to normal prostate tissues (Figure 1D). Moreover, the expression level of GLI-1 was also higher in prostatic adenocarcinomas and mainly detected in the nuclei and cytoplasm of PC cells (indicated by arrows) (Figure 1B; Figure S1). In addition, both SHH and GLI-1 were also detected in the stromal cells adjacent to malignant prostate epithelium (indicated by arrow heads; Figure 1C). These data suggest that the increase of SHH and GL1–1 expression levels in malignant epithelial cells and the stromal compartment of PC may promote the development of aggressive phenotypes during PC progression to advanced disease state.

### GDC-0449 synergizes with docetaxel in inhibiting the proliferation of human PC cells

**Figure 2:**
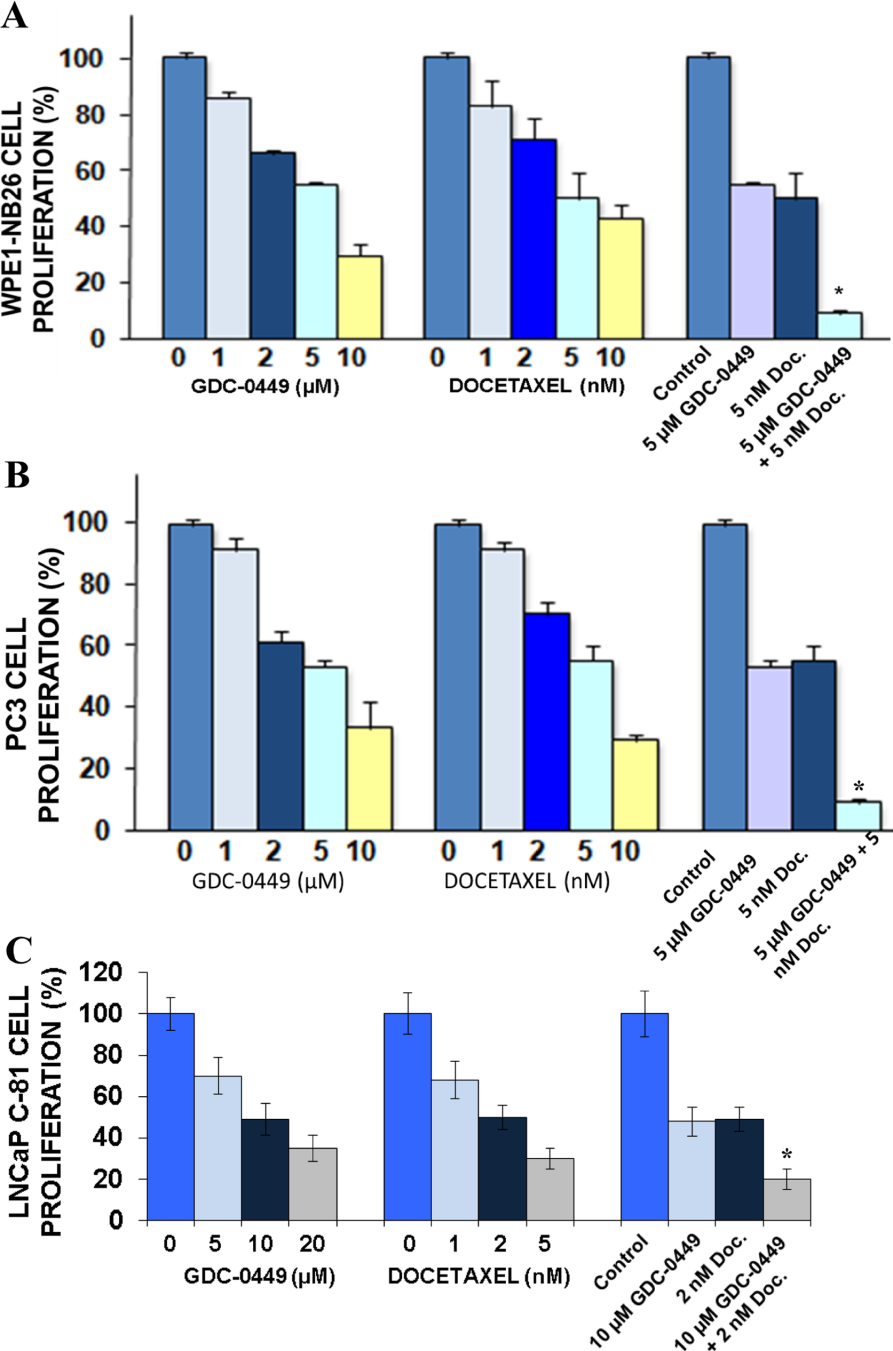
Anti-proliferative effect induced by GDC-0449 and docetaxel on PC cells **(A)** WPE1-NB26, **(B)** PC3 and **(C)** LNCaP C-81 cells were untreated (control) or treated with different concentrations of GDC-0449 and docetaxel (Doc.), alone or in combination, for two days and the cell proliferation was evaluated by MTT assay. Values shown were relative to the respective control. Data represents mean ± SE of *n* = 2 × 3. *p < 0.0001 indicates a significant difference between the anti-proliferative effect induced by a treatment with 5 μM GDC-0449 plus 5 nM docetaxel *versus* individual drugs.

ED_50_ of GDC-0449 and docetaxel were determined by MTT assay, and PC cells were treated with the sequential doses of GDC-0449 and docetaxel, alone or in combination, to determine whether GDC-0449 could enhance the antiproliferative effects of docetaxel. The results from cell proliferation assay have indicated that an increased concentration of 1–10 μM GDC-0449 or 1–10 nM docetaxel inhibited the proliferation of WPE1-NB26, LNCaP C-81 and PC3 cells (Figure 2) in a dose-dependant manner. The combination of GDC-0449 and docetaxel at ED_50_ was more effective in inhibiting the WPE1-NB26, PC3 and LNCaP C-81 cell proliferation than the drugs alone (Figure 2). Interestingly, the anti-proliferative effect of GDC-0449 at 5 μM on LNCaP C-81 cells was less effective when compared to the inhibitory effect induced by this drug on highly invasive WPE1-NB26 and metastatic PC3 cells (Figure 2C *vs* 2A and 2B). This divergence between the anti-proliferative effects of GDC-0449 might be due to the differences between the basal SHH expression levels detected in these PC cells (Figure S2). Moreover, the data from fluorescence-activated cell sorting (FACS) analyses have also revealed that the treatment of PC3 cells with increasing concentration of 1–10 μM GDC-0449 was accompanied by a blockade of PC3 cells in the G_1_ phase while 5 nM docetaxel induced a blockade of PC3 cells in G_2_M phase of cell cycle (Table 1). In addition, a combination of 5 μM GDC-0449 and 5 nM docetaxel induced a greater reduction of PC3 cell population in synthesis (S) phase through an arrest in G_1_ and G_2_M phases of the cell cycle relative to individual drugs (Table 1).

**Table 1:** Effect of GDC-0449 and docetaxel on PC3 cell cycle progression

Drug concentration	% of cells in cell cycle phase		
	G1	S	G2/M
Control	54 ± 1	28 ± 1	18 ± 1
1 μM GDC-0449	59 ± 1	24 ± 1	18 ± 1
2 μM GDC-0449	67 ± 2	19 ± 1	15 ± 1
5 μM GDC-0449	72 ± 2	14 ± 1	14 ± 2
10 μM GDC-0449	78 ± 4	8 ± 2	14 ± 1
5 nM docetaxel	48 ± 2	22 ± 1	30 ± 2
5 μM GDC-0449 + 5 nM docetaxel	67 ± 3	8 ± 2	25 ± 1

### GDC-0449 enhances the apoptotic effects induced by docetaxel on PC cells

**Figure 3:**
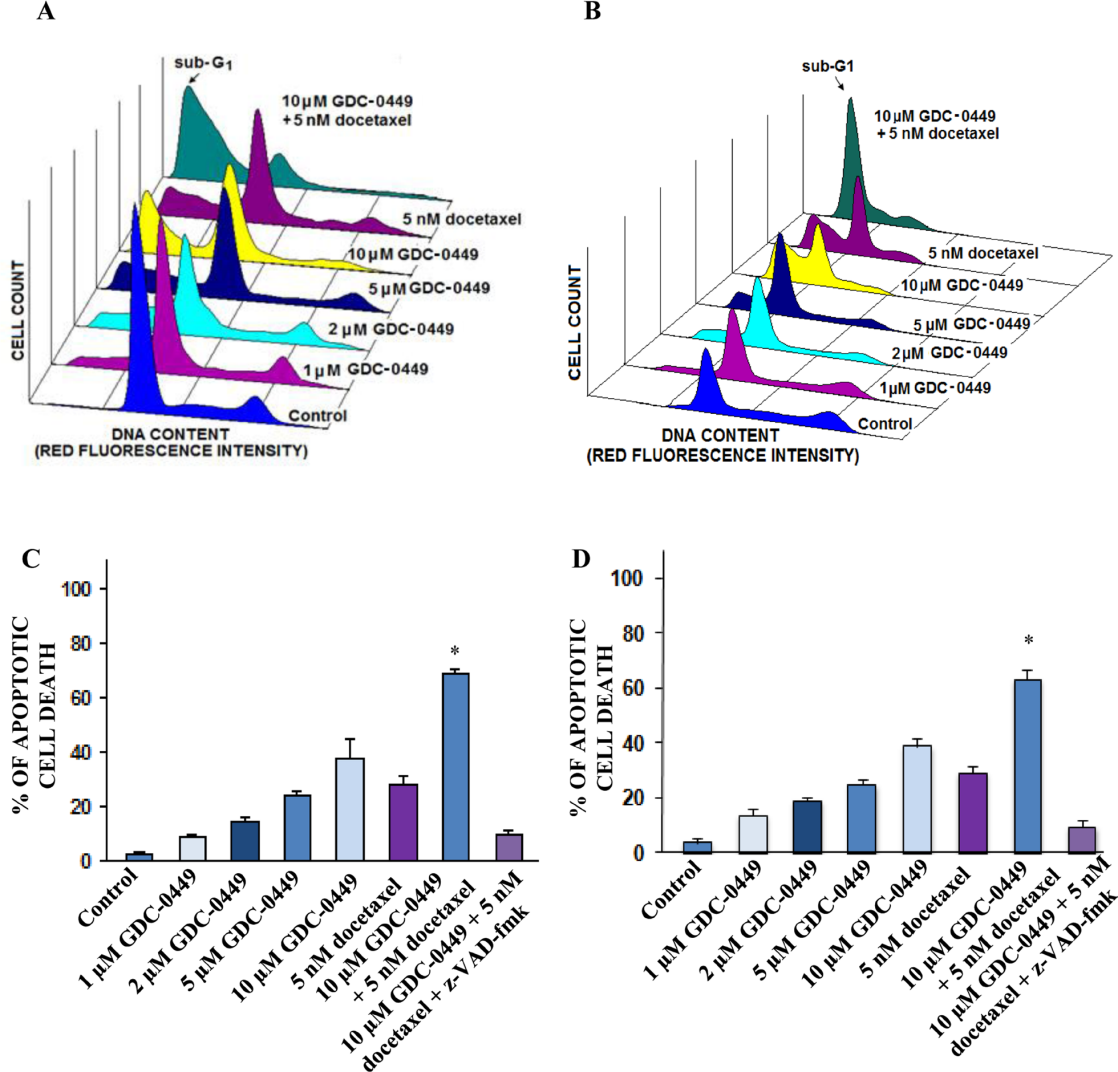
FACS analyses of apoptotic effects induced by GDC-0449 and docetaxel, alone or in combination, on PC cells PC cells were untreated (control) or treated with the indicated concentrations of GDC-0449, alone or in combination with 5 nM docetaxel in the presence or absence of broad caspase inhibitor, z-VAD-fmk for 4 days, and the apoptotic cell death was analyzed by FACS. FACS profiles obtained for **(A)** WPE1-NB26 or **(B)** PC3 cells untreated (control) or treated with 1–10 μM GDC-0449 and 5 nM docetaxel, alone or in combination, showing the apoptotic cell population in the sub-G1 phase. The panels show the apoptotic effects induced by the tested agents on **(C)** WPE1-NB26 or **(D)** PC3 cells that are expressed as the percentage of apoptotic PC cells compared to untreated PC cells (control). *p < 0.05, indicates a significant difference between the apoptotic effect induced by 10 μM GDC-0449 plus 5 nM docetaxel *versus* individual drugs.

**Figure 4:**
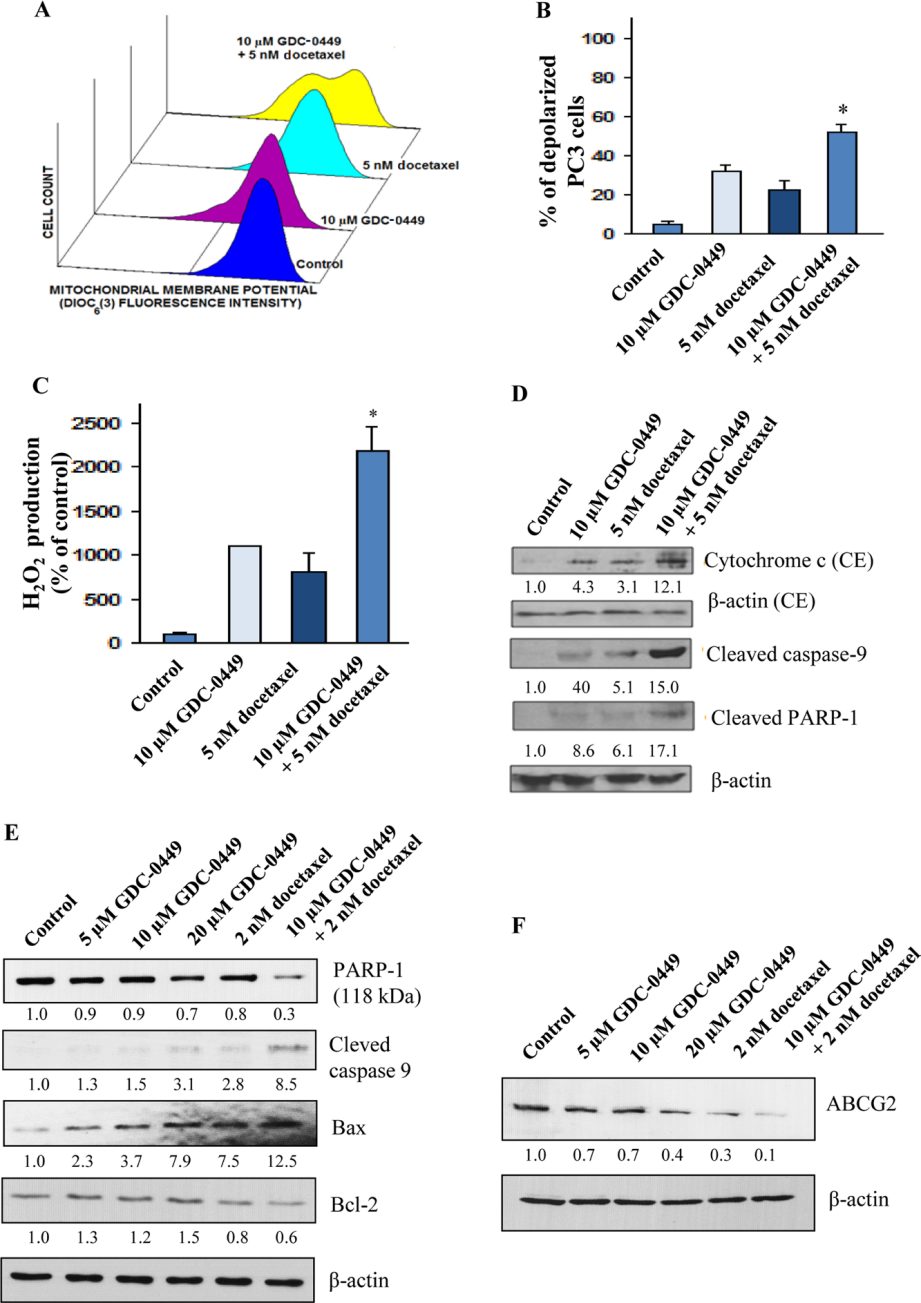
Stimulatory effect induced by GDC-0449 and docetaxel on mitochondrial membrane potential depolarization, production of reactive oxygen species and activation of caspase pathway in PC cells The PC3 cells were untreated (control) or treated with 10 μM GDC-0449 and 5 nM docetaxel, alone or in combination, over a four days time periods. After the treatments, PC3 cells were prepared by staining with 40 nM DIOC_6_ (3) for analyses of mitochondrial membrane potential by flow cytometry. **(A)** Representative profiles of stimulatory effect induced by 10 μM GDC-0449 and 5 nM docetaxel, alone or in combination, on mitochondrial membrane potential in PC3 cells are shown. **(B)** Plots showing the percentage of depolarized PC3 cells induced by a treatment with 10 μM GDC-0449 and 5 nM docetaxel, alone or in combination. **(C)** Plots showing the stimulatory effect induced by a treatment with 10 μM GDC-0449 and 5 nM docetaxel, alone or in combination, on the intracellular H_2_O_2_ production relative to untreated PC3 cells. *p < 0.05, indicates a significant difference between the stimulatory effect induced by a treatment with 10 μM GDC-0449 plus 5 nM docetaxel *versus* individual drugs on the percentage of depolarized PC3 cells and intracellular H_2_O_2_ production. **(D)** Western blot analyses of the expression levels of cytosolic cytochrome *c* and cleaved caspase-9 and PARP fragment (~85 kDa fragment) detected for PC3 cells untreated (control) or treated with 10 μM GDC-0449 and 5 nM docetaxel, alone or in combination, during four days. The relative band intensity ratios of cytochrome c, cleaved caspase-9 and cleaved PARP-1 were calculated by densitometric analyses on films and given under the respective blots. **(E)** LNCaP C-81 cells were plated in regular culture conditions for three days followed by the treatment with GDC-0449 and docetaxel, alone or in combination, for another two days. At the end of 48 hours cells were harvested and used for western blot analyses. **(F)** LNCaP C-81 cells were cultured in regular culture medium for three days, and maintained in the presence or absence of GDC-0449 and/or docetaxel for another two days. At the end of experimental period, cells were harvested and analyzed for PARP1 (Full-length PARP of ~118 kDa protein), cleaved caspase-9, Bax, Bcl-2 and ABCG2 expression levels. β-actin was detected as loading control. The relative band intensity ratios of Full length PARP-1, cleaved caspase-9, Bax, Bcl2 and ABCG2 were calculated by densitometric analyses on films and given under the respective blots.

The FACS analyses of the apoptotic PC cell population in the sub-G1 phase have indicated that 10 μM GDC-0449 or 5 nM docetaxel was effective in inducing the apoptotic effects on WPE1-NB26 and PC3 cells (Figures 3A–3D). Furthermore, a combination of 10 μM GDC-0449 and 5 nM docetaxel had a higher apoptotic effect on WPE1-NB26 and PC3 cells than the drugs alone; which was also significantly inhibited in the presence of broad caspase inhibitor, z-VAD-fmk at 50 μM (Figures 3A–3D). We further examined the effect of GDC-0449 and docetaxel on free radical generation and mitochondrial membrane potential (Figure 4) in PC3 cells. The results from FACS analyses have shown that the treatment of PC3 cells with 10 μM GDC-0449 plus 5 nM docetaxel was also accompanied by a greater depolarization of mitochondrial membrane (Figures 4A and 4B) and increase of intracellular H_2_O_2_ production (Figure 4C).

Western blot analysis further indicated that GDC-0449 and docetaxel significantly increased the expression levels of cytochrome c, cleaved caspase 9 and cleaved PARP in PC3 cells (Figure 4D). Similarly, GDC-0449 treatment significantly increased expression levels of cleaved caspase 9 and Bax protein in LNCaP C-81 cells (Figure 4E). We have also observed a decrease of the expression level of Bcl-2 and total PARP 1 (full length 118 kDa) proteins after the drug treatment of LNCaP C-81 cells. The results have also demonstrated that the treatment of LNCaP C-81 cells with GDC-0449 and docetaxel was accompanied by a decrease of the expression level of ATP-binding cassette sub-family G member 2 (ABCG2) protein (Figure 4F).

### GDC-0449 inhibits the self-renewal capability of SP PC cells expressing higher levels of stem cell markers and multidrug resistant protein

**Figure 5:**
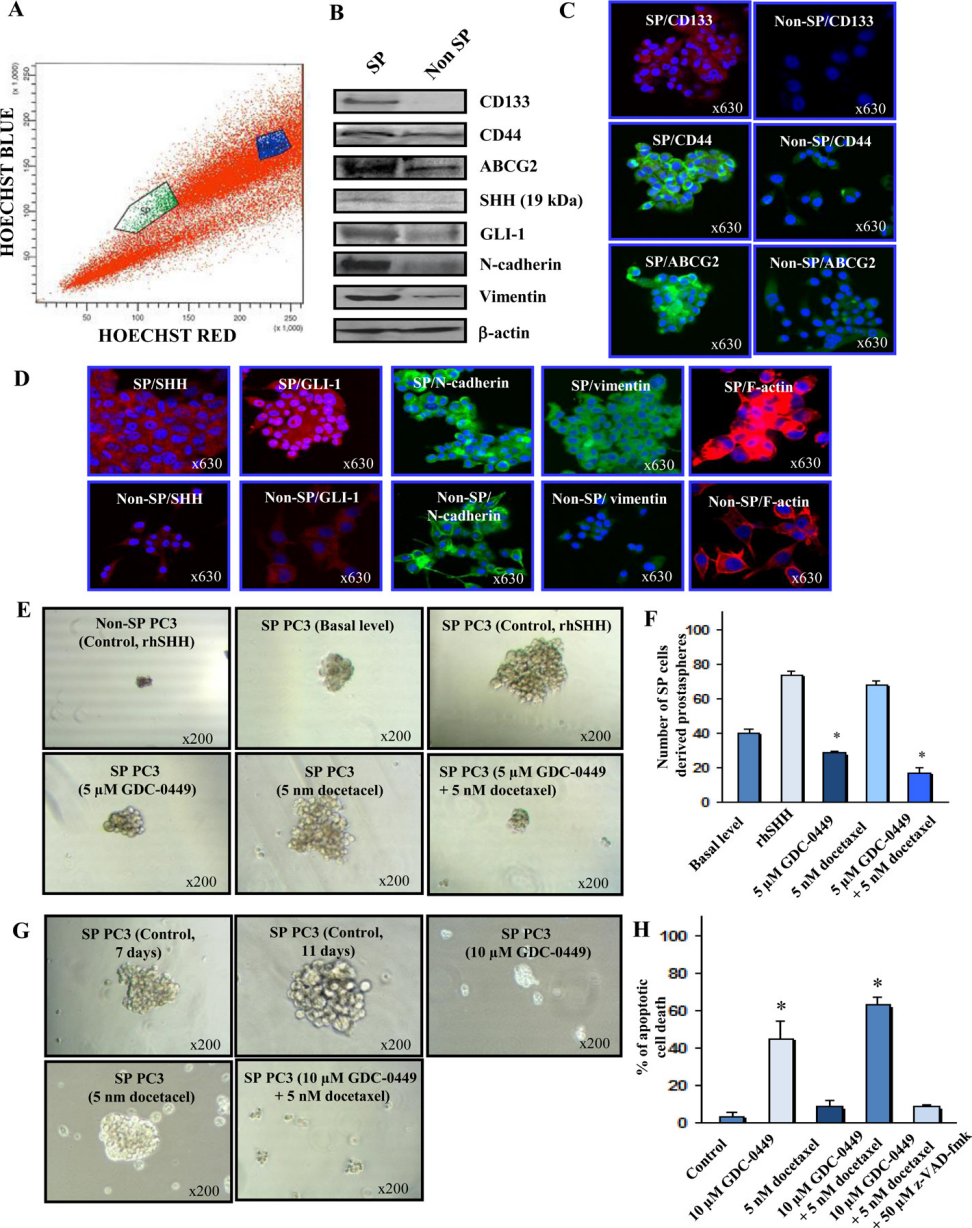
Characterization of phenotypic and functional features of SP and non-SP cell fractions from metastatic and AI PC3 cells and cytotoxic effects induced by GDC-0449 and docetaxel on SP PC3 cells **(A)** Representative data of the Hoechst dye efflux profile obtained after staining of parental PC3 cell line with fluorescent Hoechst dye showing the SP cell subpopulation (green) and non-SP fraction (blue) detected in the total mass of PC3 cells. **(B)** Western blot analyses of expression levels of prostatic stem cell-like markers (CD133 and CD44, multidrug transporter ABCG2), SHH, GLI-1, mesenchymal associated molecules (N-cadherin and vimentin) and β-actin detected in SP cells *versus* non-SP cell fraction isolated from parental PC3 cell line. **(C)** and **(D)** Representative pictures of the immunofluorescence analyses of the expression levels and cellular localization of markers in SP and non-SP PC3 cells shown at the original magnification of × 630. **(E)** Representative pictures of the dense prostaspheres formed by SP PC3 cells without or after a treatment with rhSHH as compared to diffuse, abortive and very small aggregates formed by SHH-stimulated non-SP PC3 cells. Moreover, the prostaspheres derived from PC3 cells untreated or treated with 5 uM GDC0449 and 5 nM docetaxel, alone or in combination, are shown at a similar magnification of × 200. **(F)** The self-renewal capability of SP cells versus the non-SP cell fraction from PC3 cells was estimated based on their ability to form the non-adherent aggregates in serum-free culture conditions under ultra-low attachment plate (Corning, Invitrogen). The quantitative data of the number of prostaspheres formed by the SP PC3 cell fraction without (basal level) or after a treatment with exogenous rhSHH in the absence (control) or presence of 5 μM GDC-0449 and 5 nM docetaxel, alone or in combination, are also shown. *p < 0.05 indicates a significant decrease in the number of prostaspheres formed by rhSHH-stimulated SP PC3 cells in the presence of 5 μM GDC-0449 alone or in combination with 5 nM docetaxel *versus* untreated rhSHH-stimulated SP PC3 cells. **(G)** Representative pictures of the disintegration effects induced by 10 μM GDC-0449, alone or in combination with 5 nM docetaxel, on prostaspheres derived from SP PC3 are shown at a similar magnification of × 200. **(H)** The quantitative data of the apoptotic effect induced by a treatment with 10 μM GDC-0449, alone or in combination with 5 nM docetaxel, on SP PC3 cells. *p < 0.05 indicates a significant increase in the number of apoptotic cell death induced by 10 μM GDC-0449, alone or in combination with 5 nM docetaxel, on SP PC3 cells relative to untreated SP PC3 cells.

FACS analyses have shown the presence of a small subset of SP cells representing about 0.6% of the total number of PC3 cells (Figure 5A). SP PC3 cells expressed higher levels of stem cell-like markers (CD133, CD44), multidrug transporter (ABCG2), hedgehog cascade (SHH and GLI-1) and mesenchymal markers (N-cadherin and vimentin), relative to the non-SP PC3 cell fraction (Figures 5B–5D). SP PC3 cells also displayed a greater prostasphere-forming ability than non-SP PC3 cells under serum free-medium containing rhSHH in ultra-low attachment plate (Figure 5E). Moreover, we have observed that 5 μM GDC-0449 or 5 nM docetaxel, alone or in combination, significantly inhibited the number and size of prostaspheres formed by rhSHH-stimulated SP PC3 cells while docetaxel alone had no significant effect (Figures 5E and 5F). Of therapeutic interest, a treatment of prostaspheres derived from SP PC3 cells with 10 μM GDC-0449, alone or in combination with 5 nM docetaxel, for 4 days was also accompanied by their disintegration (Figure 5G). Furthermore, a treatment of SP PC3 cells with 10 μM GDC-0449, alone or in combination with 5 nM docetaxel for 4 days, was also effective at inducing the apoptotic effect on these immature PC cells (Figure 5H).

### The combination of GDC-0449 and docetaxel displays greater anti-tumor effects than individual drugs on PC-3 xenografts

**Figure 6:**
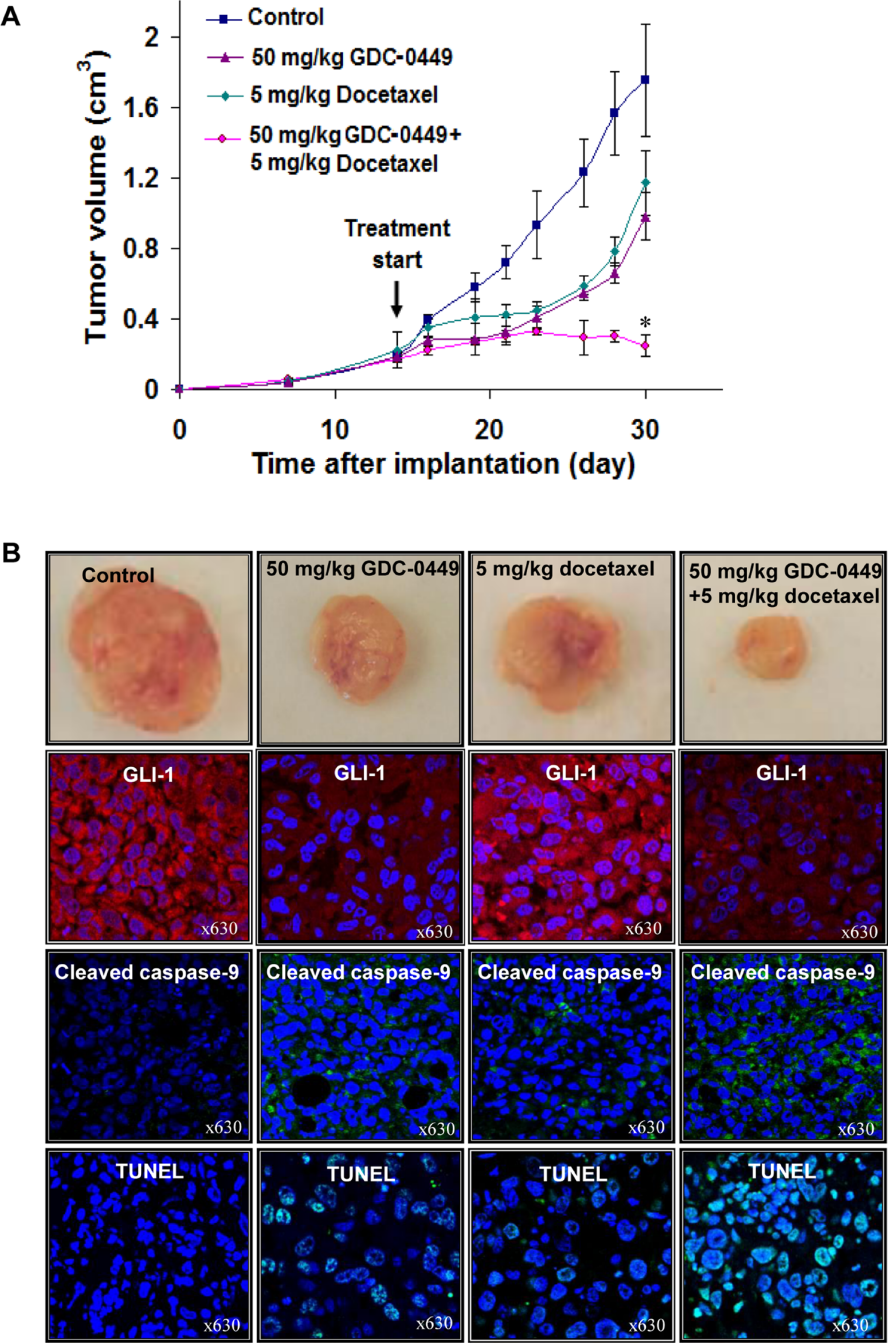
*In vivo* determination of the growth inhibitory effects induced by GDC-0449 and docetaxel on PC3 cell xenografts PC3 cells (3 × 10^6^/50 μl culture medium mixed with an equal amount of BD Matrigel™ in a final volume of 100 μl) were subcutaneously implanted in athymic nude mice. The treatment of mice with diluent (control mice) or 50 mg/kg GDC-0449 and 5 mg/kg docetaxel, alone or in combination, was initiated when the tumor volumes reached a size of about 0.2 cm^3^. **(A)** Data of tumor volumes expressed as mean +/− SE obtained for mice treated with indicated drugs *versus* control mice in function of time after implantation. *p < 0.05, indicates a significant difference between the growth inhibitory effects induced by 50 mg/kg GDC-0449 plus 5 mg/kg docetaxel *versus* individual drugs. **(B)** Pictures of PC3 xenograft tumors after 30 days of implantation obtained without (control) or after a treatment with 50 mg/kg GDC-0449 and 5 mg/kg docetaxel, alone or in combination. Moreover, the data from immunohistofluorescence analyses of the expression of GLI-1 (red staining) and cleaved caspase-9 fragment (green staining) and DNA fragmentation by TUNEL assays and counterstaining with DAPI (green/blue; hybrid cyan) in PC3 xenograft tumor specimens untreated or treated with 50 mg/kg GDC-0449 plus 5 mg/kg docetaxel, alone or in combination, are also shown at a similar magnification of × 630.

An *in vivo* xenograft study was undertaken to determine the therapeutic effect of GDC-0449 and docetaxel, alone or in combination. The results have indicated that 50 mg/kg GDC-0449 or 5 mg/kg docetaxel was effective at inhibiting the tumor growth of PC3 xenografts as compared to untreated PC3 xenografts in athymic nude mice (Figure 6A). Furthermore, a combination of 50 mg/kg GDC-0449 and 5 mg/docetaxel had a greater tumor inhibitory effect on PC3 xenograft without significant toxicity as compared to individual drugs (Figure 6A).

### Combined treatment of GDC-0449 and docetaxel inhibits the expression of GLI-1 and apoptosis in PC-3 xenograft tumors

The immunohistofluorescence analyses of PC3 tumor specimens have indicated that 50 mg/kg GDC-0449, or 5 mg/kg docetaxel alone, significantly inhibited the GLI-1 expression in PC3 xenografts as indicated by a decrease of GLI-1-positive red staining relative to untreated PC3 tumors used as control. Moreover, the combined treatment of GDC-0449 and docetaxel was more effective than the drugs alone in inducing the caspase-9 activation and DNA fragmentation in PC3 xenografts (Figure 6B). Inversely, the amount of cleaved caspase-9-positive green staining is significantly increased and positively correlated with TUNEL-positive cells in GDC-0449 and docetaxel treated groups compared to control mice.

## DISCUSSION

The microtubule-targeting agent, docetaxel is the first-line chemotherapeutic drug used for the treatment of metastatic CRPC [7]. However, the patients often develop therapeutic resistance to docetaxel-based treatment and have fewer treatment options. A growing body of evidence suggests that an aberrant activation of SHH signaling cascade is involved in the progression and development of metastatic and AI PC [12, 41]. In parallel, it has been shown that the malignant transformation of prostate-resident adult stem/progenitor cells into PC stem/progenitor cells leads to the development of aggressive, AI and recurrent cancers [12, 33]. Furthermore, these PC stem/progenitor cells are relatively resistant to both ADT and docetaxel-based chemotherapies, suggesting that their expansion may drive disease relapse of PC patients observed in the clinics [12, 32, 34]. In spite of significant recent progress, the anticarcinogenic effects induced by a combination of docetaxel and GDC-0449 on AI and metastatic PC is relatively unknown. Therefore, the present study has been carried out to investigate the anti-carcinogenic effects induced by a combined treatment of GDC-0449 and docetaxel on AI PC cells both *in vitro* and *in vivo*.

Under physiological conditions, the transmembrane protein Ptch1 inhibits the seven-span transmembrane protein Smoothened (Smo). Upon binding of hedgehog (HH) ligands to the Ptch1 relieves Smo which in turn activate GLI-1 transcription factors for the activation of downstream targets such as GLI-1, PTCH, myc, cyclin D1 and Bcl2 [42, 43]. Further, it has been shown that SHH-mediated signaling pathway directly exports the GLI-1 transcription factor enabling its cytoplasmic to nuclear shuttling [42, 44]. In agreement with the previous observations [14, 17, 45, 46], higher cytoplasmic SHH and the nuclear GLI-1 expression levels in prostatic adenocarcinomas tissues suggest that the sustained activation of sonic hedgehog cascade may contribute to the PC development and progression to locally invasive, aggressive and AI PC. Importantly, the immunohistochemical analyses have indicated that the increased expression levels of SHH and GLI-1 frequently occur in prostatic adenocarcinoma tissue specimens relative to non-malignant prostate tissues (Figure 1). Moreover, SHH and GLI-1 expression were also up-regulated in tumor-associated stromal cells in PC tissue specimens (Figure 1). In addition, the expression level of SHH was also higher in PC cells when compared to immortalized normal prostate epithelial cells (Figure S2). These data are in agreement with earlier studies that have revealed an increase of expression levels of hedgehog signaling components (SHH, patched (PTCH) receptor, smoothened (SMO) co-receptor, GLI-1 and/or GLI-2 transcription factors) were detected in some cases of high-grade prostatic intraepithelial neoplasia (PINs), PC tissues and bone metastases relative to normal prostate and adjacent non-malignant human prostate tissues [12, 13, 15–19]. More specifically, the data from immunohistochemical analyses of SHH, PTCH and GLI-1 in 155 cases of PC have indicated that the enhanced expression of these proteins was significantly associated with poor prognostic parameters, including a larger tumor size, high serum prostate-specific antigen (PSA) levels, high Gleason score, perineural invasion and anatomic stage/prognostic groups (ASPG) [18]. Moreover, it has been reported that the enhanced expression and aberrant activation of hedgehog signaling elements in PC epithelial cells and tumor-associated stromal cells may be induced through ligand-dependent autocrine and paracrine mechanisms [12, 14, 17, 38]. A shifting toward the autocrine stimulation of hedgehog cascade in PC epithelial cells has been noted during PC progression to invasive and AI disease conditions [17]. In addition, it has also been observed that the up-regulation of hedgehog signaling molecules (SHH, PTCH1, SMO, GLI-1 and GLI-2) expression levels was observed in PC patient tissue specimens who received ADT, or both ADT and docetaxel [47, 48]. Altogether, these data suggest an important role for the uncontrolled activation of the hedgehog pathway in PC cells as well as stromal cells in their tumor microenvironment during the PC development and progression to more aggressive and therapy-resistant phenotype.

Of therapeutic interest, our results have also shown that the inhibition of hedgehog pathway with GDC-0449 suppressed the growth (Figure 2) and invasiveness (Figures S3A and S3B), and induced the apoptosis in highly invasive WPE1-NB26 and metastatic and AI LNCaP C-81 and PC3 cells *in vitro* (Figures 3 and 4). Further, we also evaluated the protein expression levels of ABCG2, a multidrug transporter protein which can act as a cellular defense mechanism by effusing the drug out of the cells. The decreased level of ABCG2 protein in our study supports the use of GDC-0449 in combination with docetaxel for therapeutic enhancement. The GDC-0449 was also effective at increasing the anti-proliferative, anti-invasive and apoptotic effects induced by current chemotherapeutic drug, docetaxel on WPE1-NB26 and PC3 cells through an arrest in G_1_ and G_2_/M phases of the cell cycle and mitochondrial-dependent caspase activation (Figures 2 and 4). Importantly, GDC-0449 also inhibited the self-renewal capacity of CD133^+^/CD44^high^/ABCG2^high^ SP cells with stem cell-like properties and expressing high levels of SHH, GLI-1 and EMT process-associated molecules, and induced the cytotoxic effects and reversed the resistance of SP cells to docetaxel (Figure 5). Furthermore, GDC-0449 significantly inhibited PC3 xenograft and synergized with docetaxel in inhibiting PC3 xenograft growth (Figure 6). Hence, these data indicate that a combination of GDC-0449 plus docetaxel is more efficient than individual drugs to eliminate the total mass of PC cells, including PC stem/progenitor cells, which provide critical role in treatment resistance and tumor relapse of PC patients. In support with these observations, several preclinical studies have also shown that the blockade of hedgehog signaling pathway using an inhibitor of SMO activity, such as cyclopamine or NVP-LDE-225 (Erismodegib), reduced the proliferation, invasiveness and/or metastatic potential of PC cells, and reversed chemoresistance and prevented tumor relapse after treatment cessation *in vivo* [14, 49–51]. For instance, it has been observed that the inhibition of hedgehog pathway by NVP-LDE-225 suppressed the EMT process and sphere-forming ability of CD133^+^/CD44^+^ PC stem/progenitor cells and induced the apoptosis in these immature PC cells [50]. NVP-LDE-225 was also effective at inhibiting the tumor growth of PC cells in humanized non-obese and diabetic/severe combined immune deficient and IL2 receptor gamma (NOD/SCID IL2Rγ) null mice [50]. Furthermore, it has been observed that cyclopamine was also effective at chemosensitizing paclitaxel-resistant DU145-TXR and PC3-TXR cells to paclitaxel treatment at least in part *via* the P-glycoprotein down-regulation and reducing the SP cell fraction in these cells [52]. Similarly, docetaxel-resistant PC cell subpopulation exhibiting an up-regulation of hedgehog and Notch cascades has been detected in CRPC tissue specimens from patients who received docetaxel treatment [32]. It has also been noted that the down-regulation of hedgehog and Notch cascades by small hairpin RNA (shRNA) or a treatment with cyclopamine and γ-secretase inhibitor dibenzazepine (dbz) of docetaxel-resistant DU145-DR and 22Rv1-DR cells endowed with a tumor-initiating capacity induced their apoptosis, and chemosensitized these PC cells to docetaxel *in vitro* and *in vivo* [32].

Recently clinical trials have been undertaken to establish the benefit of using hedgehog inhibitors, such as GDC-0449, in patients with aggressive, locally advanced and metastatic cancers, including PC harboring aberrant activation of hedgehog cascades. The data from phases I and II clinical trials have revealed that GDC-0449 was well tolerated and a complete or partial tumor response occurred in some patients with locally advanced and aggressive basal cell carcinomas and medulloblastoma after a treatment with GDC-0449 as monotherapy [26, 36, 53, 54]. In spite of these promising results, it had been reported that the long-term treatment with GDC-0449 alone was associated with the persistence of chronic secondary effects (fatigue, muscle spasms, weight loss, dysgeusia, alopecia, hyponatremia and nausea) and acquisition of drug resistance by cancer cells in some cancer patients. Therefore, these observations underline the importance to develop novel combination therapies by using low doses of the drug targeting hedgehog cascade, such as GDC-0449, with other cytotoxic agents to reduce side effects and prevent the development of drug resistance.

Overall, our results have indicated that orally active sonic hedgehog inhibitor GDC-0449 is effective in inducing the anti-proliferative, anti-tumoral and apoptotic effects and improving the anti-carcinogenic effects of docetaxel on PC *in vitro* and *in vivo* models. Hence, our data will support future clinical trials aiming to determine the anti-carcinogenic properties of GDC-0449, alone or in combination with anti-hormonal treatment, radiation, chemotherapy or other molecular targeted agents, for treating patients with aggressive, metastatic, AI and relapsed PC.

## MATERIALS AND METHODS

### Reagents

Keratinocyte-SFM, RPMI-1640 and all other culture materials were purchased from Life Technologies (Carlsbad, CA, USA). Fetal bovine serum (FBS), 3-(4,5-dimethylthiazol-2-yl)-2,5-diphenyltetrazolium bromide (MTT), 3′,3′-dihexyloxacarbocyanine iodide (DiOC_6_(3)) 2′,7′-dichlorofluorescein diacetate (DCFH-DA), mouse monoclonal anti-vimentin (V6630) and anti-β-actin antibody (clone AC-15) were obtained from Sigma-Aldrich (St. Louis, MO). Docetaxel and GDC-0449 were purchased from LC laboratory (Woburn, MA, USA). Recombinant human SHH protein (rhSHH) was obtained from R&D Systems (Minneapolis, MN, USA). *N*-benzyloxycarbonyl-Val-Ala-Asp-fluoromethylketone (z-VAD-fmk) was from Calbiochem Corp. (San Diego, CA, USA). The anti-CD44 (HCAM, F-4), anti-cytochrome *c* (6H2) mouse monoclonal antibodies, CD133 (H-284), ABCG2 (B-25), SHH (H-160), GLI-1 (H-300), human poly (ADP-ribose) polymerase (PARP 197–214) fragment (H-250) rabbit polyclonal antibodies were obtained from Santa Cruz Biotechnology, Inc (Santa Cruz, CA, USA). Mouse monoclonal anti-E-cadherin and anti-N-cadherin obtained from BD Biosciences (Franklin Lakes, NJ, USA). The rabbit polyclonal cleaved caspase-9 antibody was from Cell Signaling Technology (Danvers, MA, USA). The phycoerythrin-conjugated monoclonal anti-CD133/2 antibody (293C3) was obtained from Miltenyi Biotec. Inc. (San Diego, CA, USA). The immPRESS™ reagent kit and 3, 3′-diaminobenzidine “DAB” substrate kit were purchased from Vector Laboratories (Burlingame, CA, USA).

### Cell lines

Human RWPE1, WPE1-NB26, LNCaP-FGC and PC3 cell lines were originally obtained from American Type Culture Collection (ATCC) (Manassas, VA, USA). RWPE1 is human prostate epithelial cells derived from the histologically normal tissue and established with transfecting a single copy of the human papilloma virus 18 (HPV-18) [55]. WPE1-NB26 cells were derived from RWPE-1 cells after exposure to N-methyl-N-nitrosourea (MNU) and established by two cycles of tumor growth in xenograft mice [56]. The tumorigenic WPE1-NB26 cells were cultured in keratinocyte serum-free medium (SFM) supplemented bovine pituitary extract and epidermal growth factor (EGF). PC-3 was established from a bone metastatic site of grade IV prostatic adenocarcinoma patient [57]. The highly metastatic, androgen-independent PC3 cells were grown in phenol red-positive RPMI-1640 culture medium containing 10% fetal bovine serum (FBS), 1% glutamine and 1% penicillin–streptomycin. LNCaP C-81 cells (passage number greater than 81) was established in our laboratory from androgen-sensitive LNCaP-FGC cells after prolonged culture in regular RPMI culture medium [58]. LNCaP C-81 cells were cultured in RPMI supplemented with 10% FBS, 1% glutamine and 1% penicillin-streptomycin. LNCaP C-81 cells exhibit the CR phenotype including functional AR expression and AI PSA secretion and grow rapidly in the steroid-deprived culture conditions. WPE1-NB26, LNCaP C-81 and PC3 cells were maintained in a 37°C incubator supplied with 5% CO_2_ and routinely tested for morphological features (Roche Diagnostics, Indianapolis, IN, USA).

### Immunohistochemical analyses

Immunohistochemical analyses of the expression levels of SHH and GLI-1 proteins were performed using tissue microarrays containing formalin-fixed and paraffin-embedded normal and adenocarcinomas of prostatic tissues (Biomax Inc. Rockville, Maryland, USA). The tissue microarrays PR954 containing duplicate cores from 36 patients with primary prostatic adenocarcinomas (Gleason scores: 6–10; stages T2-T4) and PR483 containing single core from 40 patients with localized prostatic adenocarcinomas (Gleason scores: 6–10; stages T2-T4) and normal prostate tissues obtained from autopsy from 8 patients of different ages (19–43 years). The immunohistochemical staining of tissue microarrays was carried out using the immPRESS™ reagent kit as per manufacturer recommendation (Vector Laboratories, Burlingame, CA, USA) [19, 20, 59]. Briefly, the tissue microarray slides were deparaffinized, and antigen retrieval performed with a solution of 0.01 M citrate buffer pH 6.0. After nonspecific blocking with normal horse serum, the tissue specimens were incubated with primary anti-SHH or anti-GLI-1 antibody in a humidified chamber overnight at 4°C. Subsequently, tissue arrays were washed with phosphate-buffered saline (PBS) and incubated with universal secondary anti-mouse/rabbit antibody for 30 min. After quenching endogenous peroxidase activity using 0.3% hydrogen peroxide in methanol:PBS (1:1) for 10 min, the tissue sections were stained with a solution of 3,3′-diaminobenzidine “DAB” substrate as indicated in the manufacturer's instructions.

For each tissue specimens, the intensity of immunoreactivity for SHH or GLI-1 protein was semi-quantitatively graded by a urologic pathologist (Dr. Johansson) on a 0 to +3 scale (0 = no staining, 1 + = week staining, 2 + = moderate staining and 3 + = strong staining). The percentage of PC cells that exhibited a positive staining for SHH or GLI-1 within a given tissue core was also scored on a 1 to 4 scale (1 = 0–25%, 2 = 26–50%, 3 = 51–75% and 4 = 76–100% positive cells). The score of the staining intensity and the percentage of immunoreactive PC cells were then multiplied to obtain a composite score ranging from 0 to 12. The means of composite score values obtained for SHH and GLI-1 staining in prostate adenocarcinoma samples were compared to the values for normal prostate tissues.

### Cell growth and apoptosis assays

For analyses of ED_50_ and anti-proliferative effects, WPE1-NB26, LNCaP C-81 or PC3 cells were seeded on 96-well plates at a density of 3 × 10^4^ cells per well in a culture medium containing 0.5% FBS with varying concentrations of GDC-0449, and docetaxel, alone or in combination, and the cell proliferation was then assessed after 48 h by using MTT assay [19, 20, 59]. To determine the cell cycle distribution, PC3 cells were also grown at a density of 5 × 10^5^ cells on 25 cm^2^ dishes and treated with different concentrations of GDC-0449 and docetaxel, alone or in combination, for 48 h and PC cell populations were analyzed by FACS. For analyses of apoptotic effects of tested drugs, WPE1-NB26 or PC3 cells were untreated (control) or treated with different concentrations of GDC-0449 and docetaxel or 10 μM GDC-0449 plus 5 nM docetaxel in the presence or absence of broad caspase inhibitor z-VAD-fmk at 50 μM for 4 days. The number of apoptotic PC cells detected in the sub-G_1_ population was analyzed by FACS as described previously [19, 20, 59].

### Determination of mitochondrial membrane potential, hydrogen peroxide production, cytosolic cytochrome c release and caspase activation

PC3 cells were treated with 10 μM GDC-0449 and 5 nM docetaxel, alone or in combination for four days. The adherent and floating PC cells were collected, rinsed twice with PBS and centrifuged. For determining mitochondrial membrane potential (MMP), 1 × 10^6^ PC3 cells were resuspended in 1 ml PBS containing 40 nM DiOC_6_(3) and incubated for 20 min at 37°C. The accumulation of DiOC_6_ (3), a cationic, lipophilic and fluorescent dye within the mitochondria of PC3 cells was measured by FACS analyses [19, 60].

For analyses of hydrogen peroxide (H_2_O_2_) production, PC3 cells were treated with 10 μM GDC-0449 and 5 nM docetaxel alone or in combination for 4 days, and the floating and adherent cells were collected and washed in PBS. The PC cell pellets were resuspended in 1 ml PBS containing 5 μM DCFH-DA dye, incubated at 37°C for 20 min and the intracellular H_2_O_2_ levels monitored by cytofluorometric analyses of the changes in the DCF fluorescence intensity emitted in green at a wavelength of 525 nm. In addition, the amounts of cytochrome *c* present in the cytosolic extracts and cleaved caspase-9 fragment and cleaved PARP fragment in PC3 cell lysates prepared from PC cells untreated (control) or treated with drugs were also estimated by western blot analyses as described above.

### Western blot analyses

PC3 and LNCaP C-81 cell lysates were prepared, and the protein concentrations were determined using Bio-Rad protein assay kit (Bio-Rad Inc., Hercules, CA) [19, 20, 61]. The PC cell lysates of 20–40 μg proteins were electrophoresed on 8 or 10% SDS-polyacrylamide gel under reducing conditions and transferred onto an immobilon-P PVDF membrane. The membranes were blocked with 5% non-fat dry milk in PBS for 2 h. Subsequently, the blots were washed in PBS containing 0.1% Tween, and incubated with respective primary antibody overnight at 4°C. Subsequently, the membranes were incubated with respective horseradish peroxidase-conjugated secondary antibodies (Amersham Biosciences, Piscataway, NJ) for 1 h. Antibody-antigen complexes were visualized using enhanced chemiluminescence kit (Amersham Biosciences, USA). To analyze the intensity of the bands, densitometry was conducted on the autoradiograms using ImageJ.exe program (http://rsb.info.nih.gov/). To calculate the relative protein level, each densitometer value was normalized to the corresponding b-actin protein level, then to respective controls. The ratio of control groups were considered as 1.0.

### Prostasphere-forming and disintegration assays

The parental PC3 cells (1 × 10^6^ cells/mL) were stained with fluorescent Hoechst dye at a final concentration of 2 μg/mL for 2 h at 37°C and subsequently SP and non-SP cell fractions were isolated using a FACS (Becton Dickinson Biosciences) as previously described [61–63]. The SP and non-SP cell fractions are maintained in respective serum-free medium containing exogenous EGF (10 ng/mL) plus fibroblast growth factor at 8 ng/mL before their use for experiment. The self-renewal capability of SP cells *versus* the non-SP cell fraction from PC3 cells was estimated based on their ability to form the non-adherent aggregates in serum-free culture conditions under ultra-low attachment plate (Corning, Invitrogen). For prostasphere-forming assays, 500 viable SP or non-SP PC3 cells were suspended in serum free medium with or without exogenous rhSHH (10 ng/ml) onto a 6-well plate. The prostasphere was also treated with 5 μM GDC-0449 or 5 nM docetaxel, alone or in combination. For each group, cells were plated in triplicate. After seven days of incubation, the numbers of prostaspheres formed were counted, and the representative SP PC3 cell-derived prostaspheres were photographed (Accu-scope phase-contrast microscope) at a magnification of × 200.

For the prostasphere disintegration assays, 500 viable SP PC3 cells were grown in serum-free culture conditions containing 10 ng/ml rhSHH under an ultra-low attachment plate during seven days for the formation of prostaspheres and then 10 μM GDC-0449 and 5 nM docetaxel, alone or in combination, were added to culture medium and incubated for additional four days. At day eleven, the representative pictures of disintegrated prostaspheres were photographed (Accu-scope phase-contrast microscope) at a magnification of × 200.

### Confocal microscopy analyses

Parental PC3 cells or SP and non-SP cell fractions isolated from PC3 cells by FACS as described above and were grown in sterilized cover slips for 24 h, washed with PBS, and fixed in ice-cold methanol at −20°C for 2 min. After 24 hours, the PC cells were blocked by 10% normal goat serum at 25°C for 30 min. Then, PC cells were incubated with phycoerythrin-conjugated monoclonal anti-CD133/2 antibody (293C3), rabbit polyclonal antibody directed against ABCG2 (B-25), SHH (H-160), GLI-1 (H-300), cleaved caspase-9 fragment (Asp330), cleaved PARP fragment (H-250) or mouse monoclonal antibody directed against CD44 (HCAM, F-4), N-cadherin, vimentin (V6630), cytochrome *c* (6H2) or β-actin (clone AC-15) diluted in PBS for 1 h at room temperature. After three washes with PBS, PC3 cells were then incubated with fluorescein isothiocyanate (FITC)-conjugated goat anti-mouse and/or Texas red-conjugated goat anti-rabbit secondary antibody (Jackson ImmunoResearch Laboratories, Inc., West Grove, PA) for 1 h and washed again with PBS. In addition, for F-actin staining, PC3 cells were fixed with 3.7% formaldehyde, permeabilized with 0.5% Triton X-100 and stained by using red fluorescent rhodamine phalloidin (R415; Molecular Probes, Eugene, OR, USA) for 20 min at room temperature. PC3 cell nuclei were counterstained with diamidino-2-phenylindole (DAPI), and cover slips mounted on glass slides in anti-fade Vestashield mounting medium (Vector Laboratories, Burlingame, CA). Immunofluorescence staining of PC3 cells was observed under a confocal laser scanning microscope (LSM 510, Zeiss, Gottingen, Germany) and photographed at a magnification of × 630.

In addition, immunohistofluorescence analyses of GLI-1 and cleaved caspase-9 fragment were also carried out using tumor tissue specimens from untreated or treated PC3 xenografts. The tissue slides were blocked with 10% goat serum for 30 min followed by incubation with the primary antibody against GLI-1 or cleaved caspase-9 fragment for 2 h. The slides were washed twice with PBS and processed for immunofluorescent detection as described above for the confocal microscopic analyses of fixed PC cells. Further, the terminal deoxynucleotidyl transferase dUTP nick end labeling (TUNEL) assay was also performed to detect the DNA fragmentation indicative of apoptotic cell death induced by GDC-0449 and docetaxel on tumor tissue specimens from untreated or treated PC3 xenografts. The tissue sections were incubated in the TdT reaction mixture consisting of nucleotide-labeling mix (TUNEL Label) contains fluorescein-dUTP and -dNTPs plus TdT enzyme (Roche diagnostics, IN) in a humidified chamber in the dark for 1 h at 37°C. After three washes with PBS, tissue sections were counterstained with DAPI and visualized by confocal fluorescence microscopy.

### *In vivo* study

*In vivo* experiments were performed in accordance with a protocol approved by the UNMC Institutional Animal Care and Use Committee. PC3 cells (3 × 10^6^ cells) were subcutaneously implanted on the back of male athymic nude mice in a 100 μl mixture (1:1 v/v of culture medium/matrigel). To determine the growth inhibitory effect of docetaxel and GDC-0449 alone or in combination, the treatment was initiated after the PC3 cell-derived xenografts reached a size of 0.2 cm^3^. Briefly, mice were randomly assigned into four groups of five mice per group. Control mice bearing tumors received the vehicle alone (DMSO) while other groups were treated with 50 mg/kg GDC-0449 given orally (oral gavage) once a day for five days per week and 5 mg/kg docetaxel given intraperitonally (i.p.) two days by week, for two consecutive weeks. The PC3 cells derived tumors growth was measured using a caliper for every three days throughout the experiment. At the end of these experiments, the mice were sacrificed, and the tumors were excised, weighed, and prepared for immunohistofluorescence analyses of tumor tissues.

### Statistical analyses

Statistical analyses were performed by using the Student's *t*-test to compare the results with *P* values < 0.05 indicating statistically significant between two groups. The composite scores of immunohistochemical data obtained for SHH and GLI-1 expression in non-malignant and malignant prostate tissue specimens were analyzed by using MedCalc for Windows (version 9.6.4.0.) (Ostend, Belgium). The values of composite scores were considered as continuous variables and compared using Student's two-tailed *t*-test assuming unequal variance for independent samples.

## SUPPLEMENTARY FIGURES


