# Supplementary material for: Inhibition of hedgehog signaling improves the anti-carcinogenic effects of docetaxel in prostate cancer
Source: Oncotarget. 2015 Feb 17;6(6):3887–903. doi: 10.18632/oncotarget.2932 (PMC4414161; doi:10.18632/oncotarget.2932)
Supplement: Supplementary file 1 [file oncotarget-06-3887-s001.pdf]

SUPPLEMENTARY FIGURES

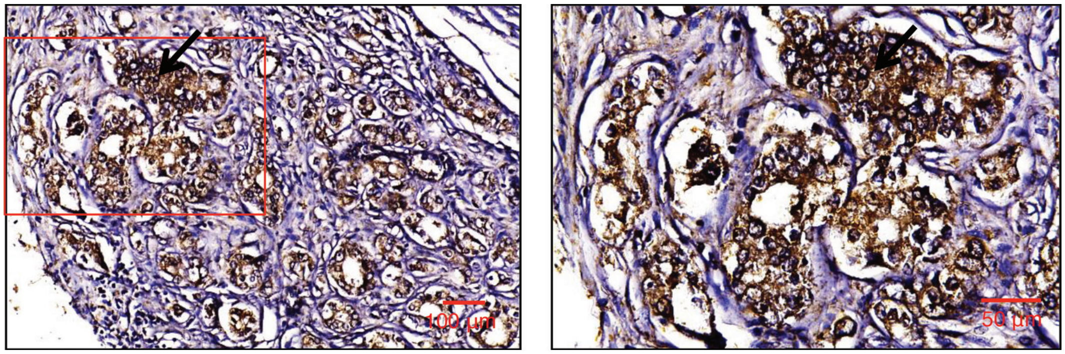

Supplementary Figure S1: Immunohistochemical analyses of GLI-1 expression levels in prostatic adenocarcinoma tissues. Immunohistochemistry staining was performed on tissue microarrays using antibodies against GLI-1. Representative micrograph of GLI-1 expression in stromal and epithelial cells of human prostatic adenocarcinoma tissues (×100). Arrow indicates the postive immunostatining for GLI-1 expression. The right panel shows a higher magnification of GLI-1 positive cells (×200).

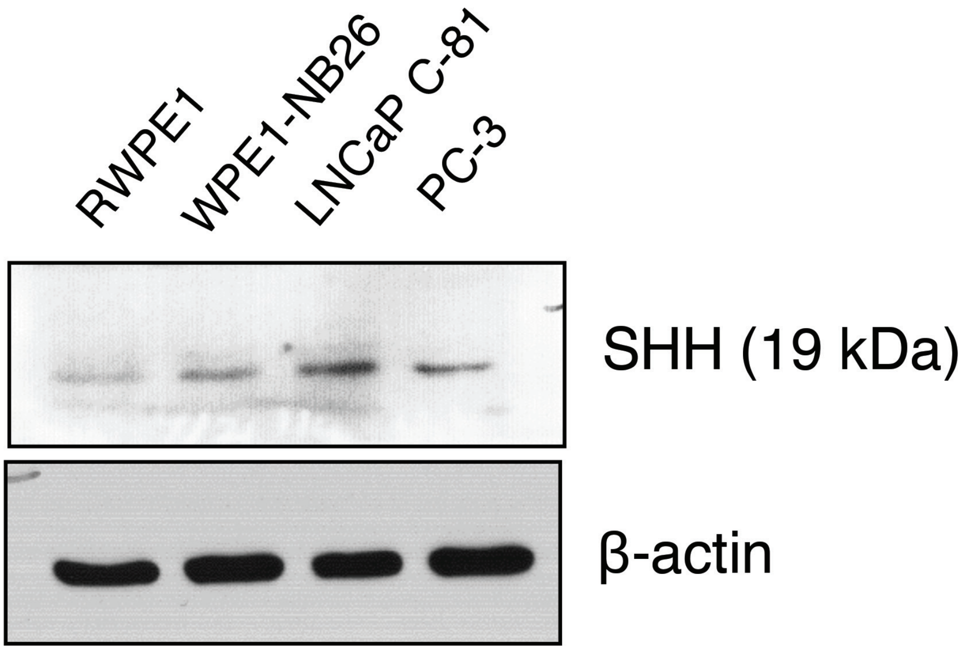

Supplementary Figure S2: Western blot analyses of SHH expression level in normal and prostate cancer (Pca) cells. PCa cells were cultured in respective culture medium for three days, followed by their maintenance in the fresh medium for another two days. At the end of five days, cells were harvested in exponential phase for determining SHH expression levels. β-actin was detected as loading control.

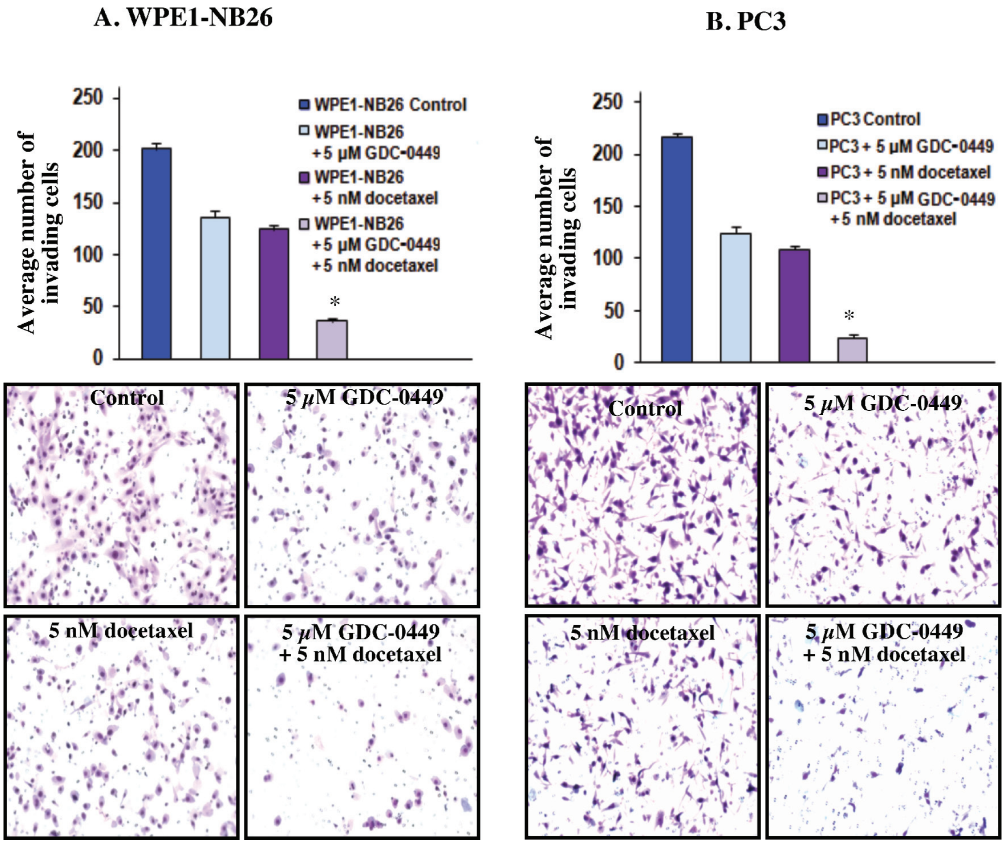

Supplementary Figure S3: Anti-invasive effects induced by GDC-0449 and docetaxel in PC cells. **(A)** WPE1-NB26 and **(B)** PC3 cells were plated on matrigel-coated membrane and treated with different concentrations of GDC-0449 and docetaxel, alone or in combination, for 24 hours. The data are presented as the average number of PC cells per field of view. $*p < 0.05$ indicates a significant difference between the inhibitory effect induced by a treatment with 5 µM GDC-0449 plus 5 nM docetaxel *versus* individual drugs on the number of invading PC cells.
